# AI4Green: An Open-Source ELN for Green and Sustainable Chemistry

**DOI:** 10.1021/acs.jcim.3c00306

**Published:** 2023-05-08

**Authors:** Samuel Boobier, Joseph C. Davies, Ivan N. Derbenev, Christopher M. Handley, Jonathan D. Hirst

**Affiliations:** † School of Chemistry, University of Nottingham, University Park, Nottingham NG7 2RD, United Kingdom; ‡ Digital Research Service, University of Nottingham, Jubilee Campus, Nottingham NG8 1BB, United Kingdom

## Abstract

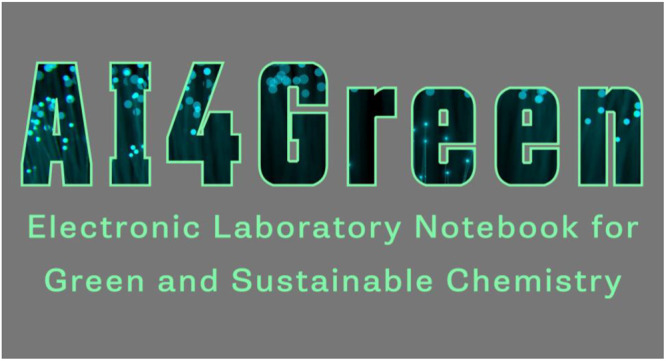

An Electronic Laboratory Notebook (ELN) combining features, including data archival, collaboration tools, and green and sustainability metrics for organic chemistry, is presented. AI4Green is a web-based application, available as open-source code and free to use. It offers the core functionality of an ELN, namely, the ability to store reactions securely and share them among different members of a research team. As users plan their reactions and record them in the ELN, green and sustainable chemistry is encouraged by automatically calculating green metrics and color-coding hazards, solvents, and reaction conditions. The interface links a database constructed from data extracted from PubChem, enabling the automatic collation of information for reactions. The application’s design facilitates the development of auxiliary sustainability applications, such as our Solvent Guide. As more reaction data are captured, subsequent work will include providing “intelligent” sustainability suggestions to the user.

## Introduction

For researchers to communicate their findings between their team and the wider scientific community, data must be shared and stored. Paper-based laboratory notebooks are traditionally used to record experiments, and little has changed over the last few decades, despite the ubiquity of digital technology. Over the past 20 years, Electronic Laboratory Notebooks (ELNs) have become more prevalent as the benefits of digitization are realized.^1^ Despite this, there remains a significant barrier to the uptake of ELNs, especially in the academic community.^2^ In 2017, a survey at a BioSistemika Webinar revealed that only 7% of respondents used an ELN in their daily laboratory routine.^2^ Another survey from the same study showed that the main barriers were the cost associated with implementing an ELN and the system’s usability.

Recently, in a comprehensive comparison of commercial and open-source ELNs, it was discovered that the majority of the 96 currently active ELNs are commercial.^3^ It was also noted that open-source codebases have the advantage that users could more directly contribute to the development of new features and have more control over the underlying software. However, there is more onus on the institution to install, host, and maintain the infrastructure. Chemotion is an open-source ELN designed for synthetic chemistry with a growing user base and a strong focus on data sharing and integrity,^4,5^ but not a particular emphasis on green and sustainable chemistry. Another open-source solution is eLabFTW, an ELN suitable for storing data from various scientific disciplines.^6^

Research data management is fundamental to scientific research.^7^ Transitioning from paper-based laboratory notebooks to ELNs is crucial for adhering to data standards when reporting and publishing studies.^8^ ELNs are also vital in making data FAIR (Findable, Accessible, Interoperable, and Reusable).^9^ ELNs allow data sharing among colleagues, institutions, and facilitate public access.^10^ Open Science^11,12^ can be enabled using ELNs, where data is curated using a standard data format, expediting data searches and preparation for machine learning, where large data sets are often required to train insightful models. Recent examples of such databases include the Open Reaction Database^13^ and the Chemotion Repository.^14^

Sustainability and reducing waste are vital considerations in laboratory-based projects. Sustainable refers to both the environmental and socio-economic impacts of a process.^15^ Making processes more sustainable is not just a requirement of government regulations. There are also the benefits of cost reductions, improved worker health and safety, and the reduction of impact on the environment.^16,17^ Current software tools for green and sustainable chemistry have recently been reviewed.^18^ ELNs also offer the opportunity for collecting data that can be used to monitor sustainability targets (such as the reduction of hazardous solvents) and share knowledge among colleagues.^19^

In this work, we present AI4Green, designed to fulfill the core functionality of an ELN for synthetic organic chemistry in academic and industry settings while encouraging green and sustainable chemistry. The software automatically presents the hazards and sustainability of an inputted reaction by calculating sustainability metrics and a color-coded assessment of solvents and reaction conditions. While the web application is open-source, the software is provided in a manner that has a low barrier to installation and hosting, has a user-friendly interface, and is easily customizable. As the number of users grows, the captured reaction data will be subsequently leveraged using machine learning to provide “intelligent” suggestions to users on improving their reactions’ sustainability.

## Implementation

**Figure 1:**
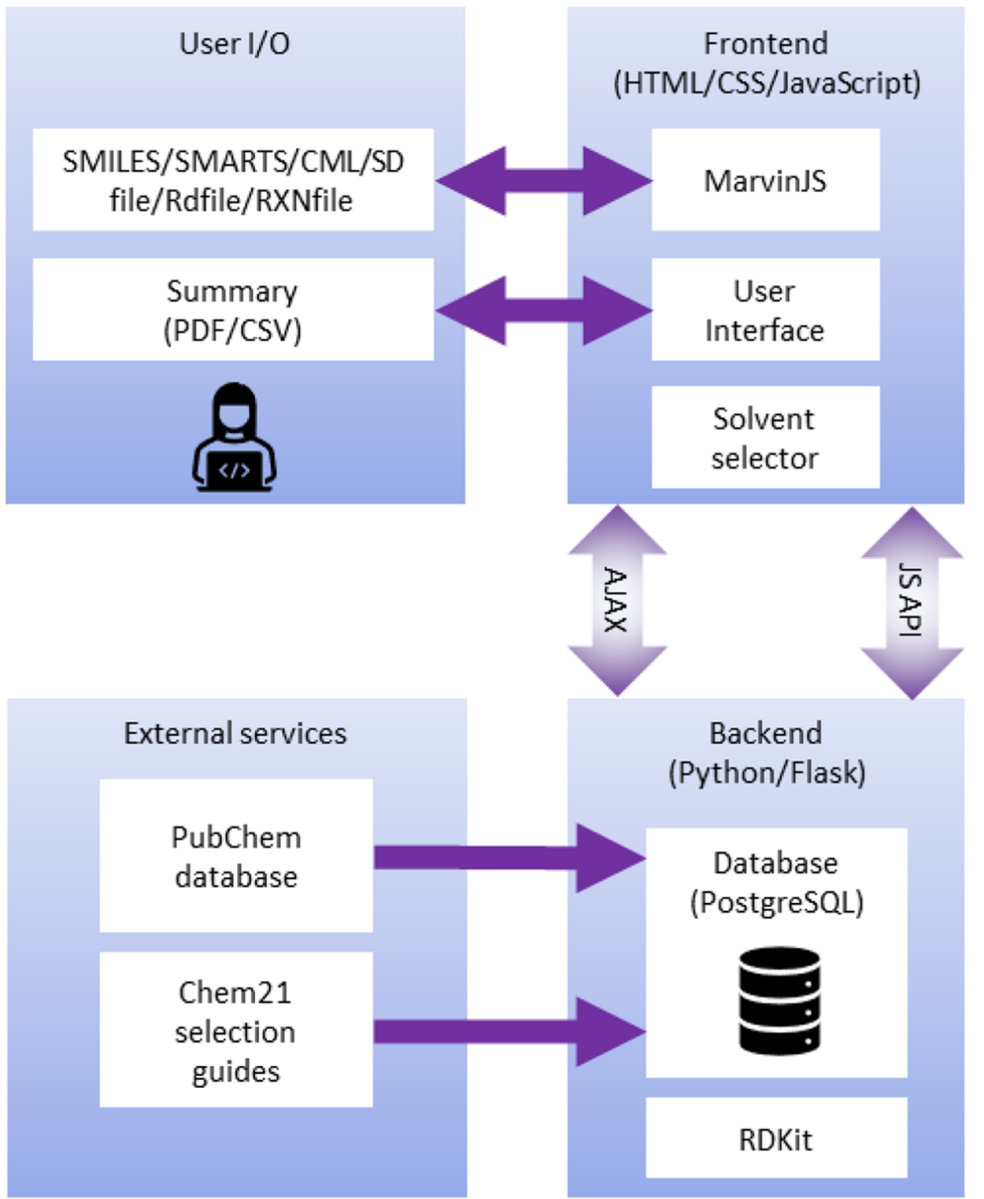
Web application architecture showing programming languages, databases and how users interact with the application.

AI4Green is a web application written in Python, JavaScript, HTML, and CSS (Figure 1). The application is hosted on the cloud and available for general use at https://ai4green.app. Alternatively, visit https://github.com/AI4Green/AI4Green for simple instructions detailing installation and hosting via Docker either locally or on an organization’s local server.

The backend server is built with Python and Flask and is linked to a Postgres relational database. The advantage of using Python for the backend is the access to many popular standard chemistry libraries, such as RDKit,^20^ and a low barrier for developers to add additional features. Flask is a well-documented and easy-to-use web application framework,^21^ and blueprints provide a clear structure to the code and facilitate expansion. Flask separates the Python backend from the JavaScript, HTML, and CSS-controlled frontend, where users input their data via MarvinJS^22^ chemical editor or in a number of formats including SMILES.^23^ JavaScript AJAX requests are used to update pages dynamically, e.g., automatically calculating green and sustainable metrics when inputting user data. A summary and sustainability report are presented back to the user which can be exported as a pdf or csv file. A single database was implemented in Postgres, constructed from compound data extracted from PubChem^24^ and CHEM21 sustainability data,^25^ to provide chemical information automatically, and separated into tables for users, workgroups, workbooks, solvents, hazards, compounds (reagents) and reactions.

## Results

### Workgroups, Workbooks, and User Types

**Figure 2:**
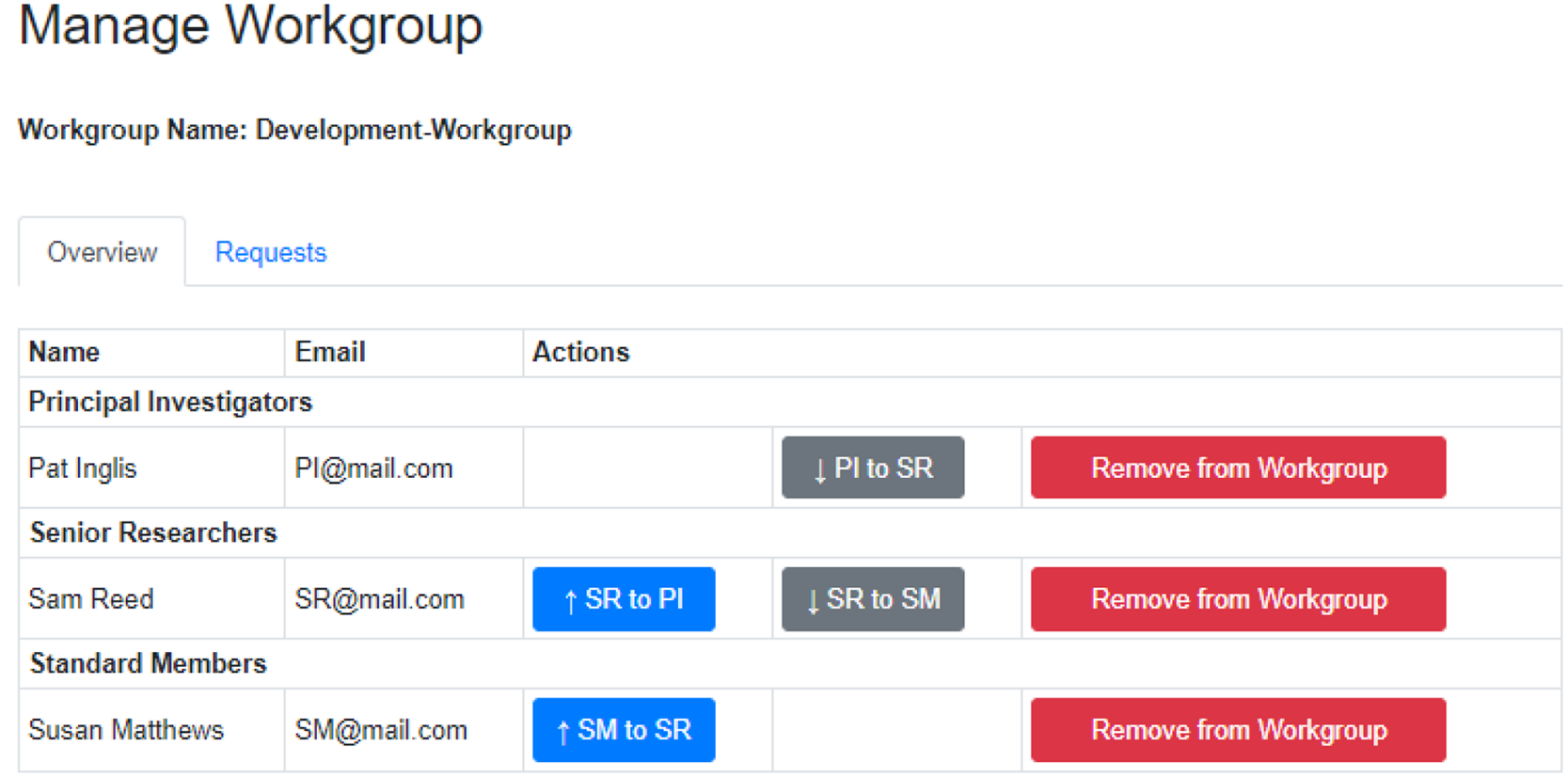
Workgroup management page where Principal Investigators can change the roles of group members and approve join requests. PI – Principal Investigator, SR – Senior Researcher, and SM – Standard Member.

AI4Green installations may have one or more admin users. These users, typically system administrators for an institution, review requests to make new Workgroups and can monitor the number of users, compounds, and reactions on the server. Users must register for an account to use AI4Green, at which point Principal Investigators, or equivalent roles, are prompted to make a Workgroup as a space for their research group; other users are directed to join the Workgroup their Principal Investigator has created. Within Workgroups, there are Workbooks that are designed to contain reactions for a specific project (Figure 2). Workgroups have three roles with different permission levels. Principal Investigators are the Workgroup owner and have full permission to create Workbooks and add or remove users from the Workgroup and any Workbooks within it. It is permissible to have two or more Principal Investigators in a Workgroup. Senior Researchers, suitable for postdoctoral researchers or equivalent, can create new Workbooks and add or remove users to these Workbooks. They have no such rights for Workgroups. Standard Members, suitable for postgraduate researchers or equivalent, have no editing rights but can request to be added to Workbooks. Using this flexible approach, a user can belong to multiple Workgroups in different roles, e.g., a Principal Investigator in one Workgroup and a Senior Researcher in another. Reactions are only shared within the same Workbook, as are any novel compounds added to the database. A user owns the reactions they create, which are also available as read-only entries to all members of the Workbook, thus enabling data sharing between team members. This is especially useful when teams are spread over multiple locations while preserving data privacy.

### Reaction Builder

The core functionality of AI4Green is the Reaction Builder. In later sections, we give further details on the different components of the Reaction Builder. Reactions can be created by navigating to a Workgroup, choosing a Workbook, and selecting “New Reaction”. The user is prompted to enter a name for the reaction which must be unique within the Workbook. In addition, a unique code is also assigned to every new reaction. Users draw their reaction into the reaction sketcher. Next, the user is prompted to fill in the Reaction Table, for example, inputting the amount of each reaction component. At this stage, further solvents and reagents can be added. Finally, the Summary Table, which contains several automatically calculated green and sustainable metrics and detailed health and safety information, is generated. The reaction is automatically saved when new changes are made and can be reloaded and edited at a later date. The Summary Table can be exported to pdf or printed for use as a risk assessment.

### Reaction Sketcher

**Figure 3:**
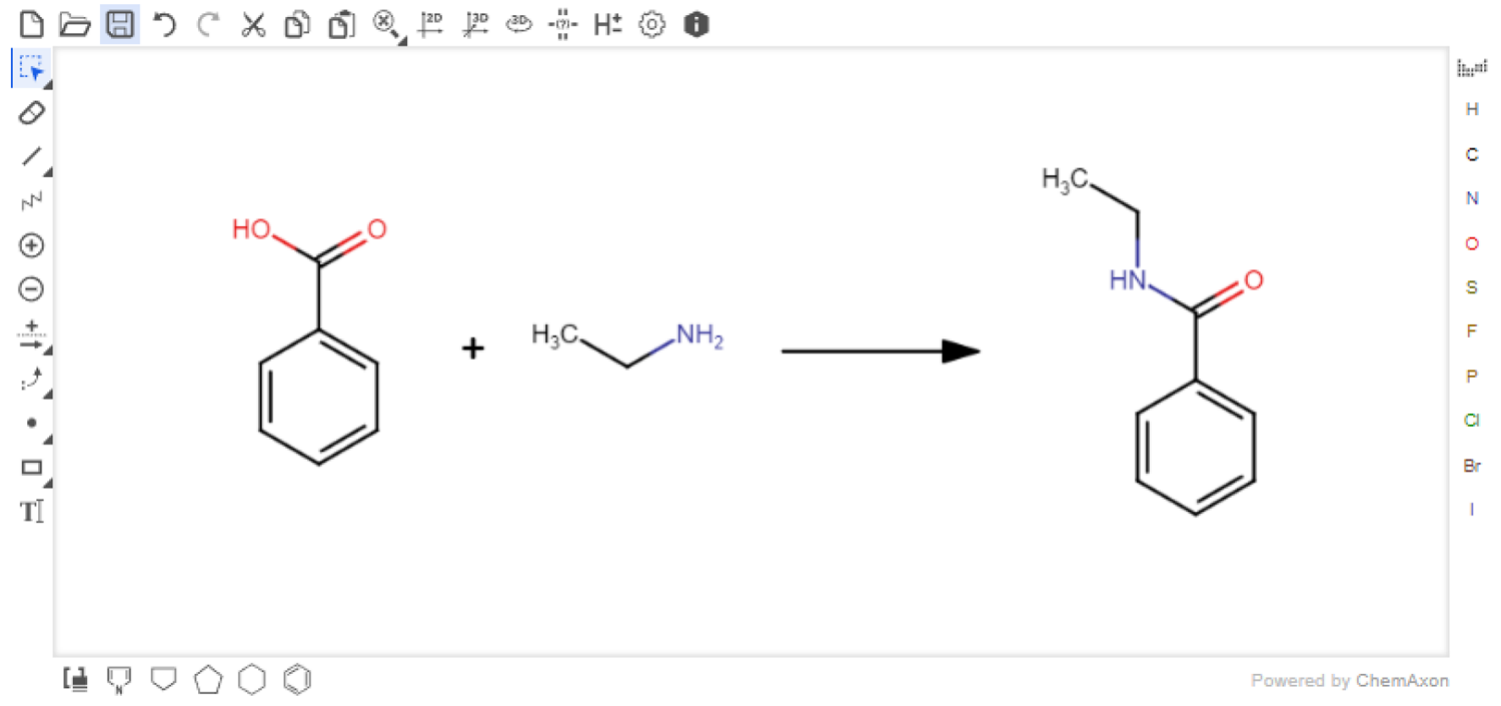
Users draw their reaction using the Marvin reaction sketcher. Reactions can also be imported in a variety of formats including SMILES.

Users must first input their reaction with the Marvin JS reaction sketcher (Figure 3). This sketcher is easy to use and well-documented.^22^ For users familiar with other sketchers, it is possible to import structures in several formats, e.g., SMILES,^23^ which are easily exported from other sketchers. Reagents or solvents above or below the arrow are not currently accepted. However, these can be added directly to the Reaction Table. When the reaction is submitted, the reaction SMILES (RXSMILES) is exported from the sketcher to the Reaction Table. The database containing information from PubChem is queried for the reactants and products in the RXSMILES to obtain density, molecular weight, and hazard codes automatically. All compound data have been collected from PubChem laboratory chemical safety sheets (LCSS). The hazard data are presented as global harmonized system of classification and labeling of chemicals (GHS) hazard codes. The hazard data are only collected from the references provided by the European Chemicals Agency (ECHA).^26^

### Reaction Table

**Figure 4:**
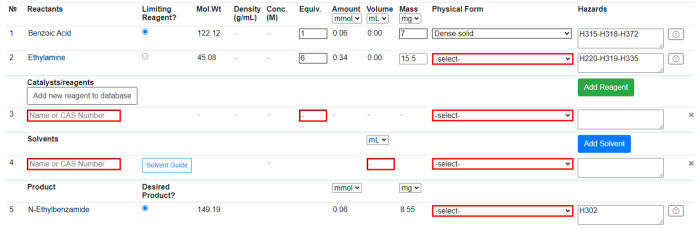
A partially complete Reaction Table. Users are directed to provide information about the reaction (highlighted in red). Reagents, solvents, and novel compounds can be added or removed. Some information such as molecular weight and hazard codes are automatically populated from the PubChem database.

The user is prompted to populate the Reaction Table (Figure 4) and provide extra information if any reactant or product is not in the database. This “Novel Compound” is saved to the database and can be reused, but only within the same Workbook. Reagents can be added from the PubChem compound database by searching name or CAS; they can also be added to the database like a Novel Compound. Solvents can also be added from a predefined list and by searching name or CAS. “Novel Solvents” can be added in the same way as “Novel Compounds”. Solvents are color-coded according to the four-tier CHEM21 classification:^25^ recommended, problematic, hazardous, and highly hazardous, providing immediate feedback to the user on the sustainability of their solvent choice. Users will then input the details of their reaction into the Reaction Table. Physical forms of all reactants, reagents, solvents, and products must be provided to assess the reaction’s risk. Additionally, limiting reagent mass and the equivalence of all other reactants and reagents are required to proceed. Any suspected incorrect data from the database can be reported to system administrators for review at any point in the procedure. There is also space to describe the experimental procedure and any observations made during the reaction.

### Summary Table

**Figure 5:**
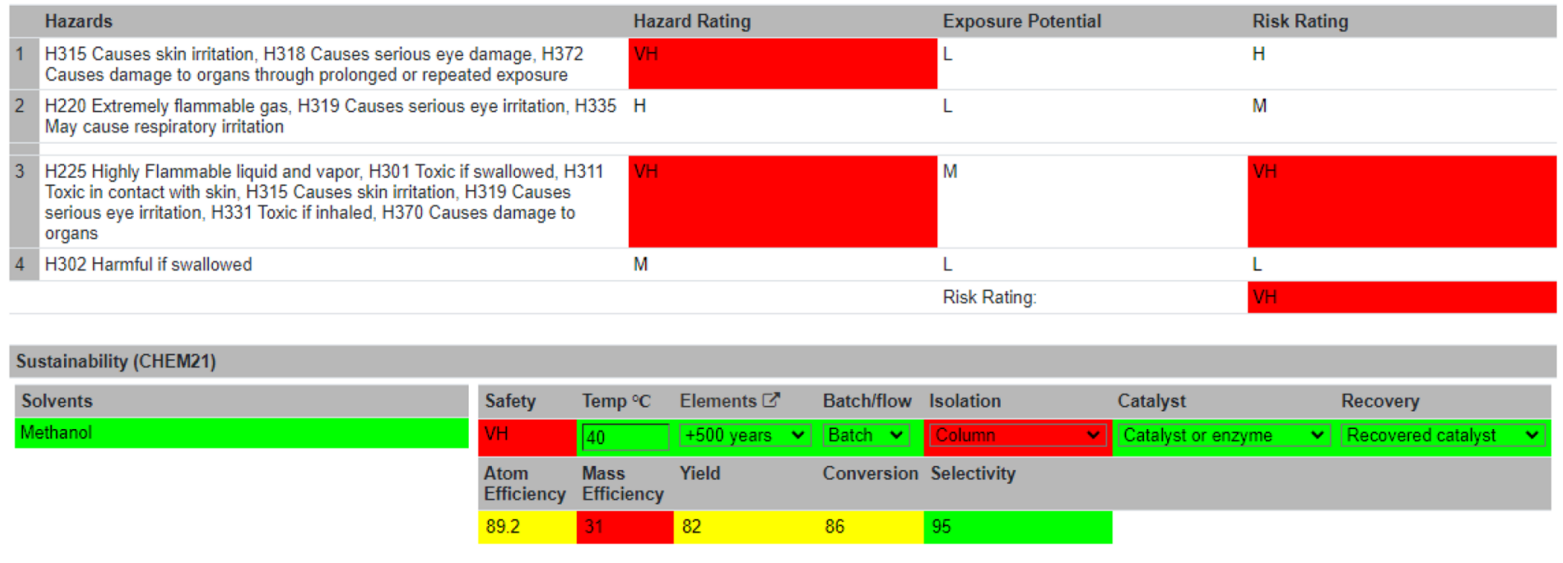
Part of the Summary Table showing information about the hazards of the reactions and various green and sustainability metrics and considerations.

With the Reaction Table complete, users are directed to the Summary Table (Figure 5). Information is automatically passed from the Reaction Table to the Summary Table. Visual assessments of the greenness and sustainability of the reaction are displayed to the users. These are either flagged as unsustainable (red) or given a traffic light system (red = not recommended/hazardous, yellow = problematic, and green = recommended). The specific colors and shades for these ratings can be altered on the accessibility page. An overall hazard rating is generated from the hazard codes, denoted as Low (L), Medium (M), Hazardous (H), or Very Hazardous (VH). The threshold of the sustainability levels of the following metrics was in accordance with the CHEM21 project.^27^ Several of these metrics are calculated automatically, like the sustainability of the chemical elements used in the reaction and the atom efficiency. Other metrics must be inputted by the user, such as the temperature of the reaction, batch or flow reaction conditions, the isolation method, the use of a catalyst, and whether that catalyst was recovered. A risk assessment section follows, which allows users to identify standard protocols, disposal of waste materials, spillage procedures, and any other risks associated with the reaction. An overall risk score can then be computed by self-assessment of the reaction’s hazards, risks, and consequences. Typically, a reaction would be performed at this point. After the reaction run, the user can return to the Summary Table and input unreacted and actual product mass. Using these inputs, four more metrics are computed: mass efficiency, yield, conversion, and selectivity. The reaction can be marked as complete and locked to further editing at this stage. For increased data integrity, reactions modifications are time-stamped in the database. Reactions may currently be searched alphabetically or by most recently created.

### Export Data

A reaction can be exported as a pdf, as some institutions may require reaction information and risk assessments to be displayed or filed. It is critical that data can be safely exported from an ELN for the longevity of that data, to move the data to a new system, and for more detailed analysis. The reactions from a Workbook can be exported as a single pdf file, multiple pdf files, or a csv file. The versatility of csv files, which are machine-readable and can be opened in programs such as Microsoft Excel, makes this file format the preferred option for data offloading.

### Sustainability Add-Ons

**Figure 6:**
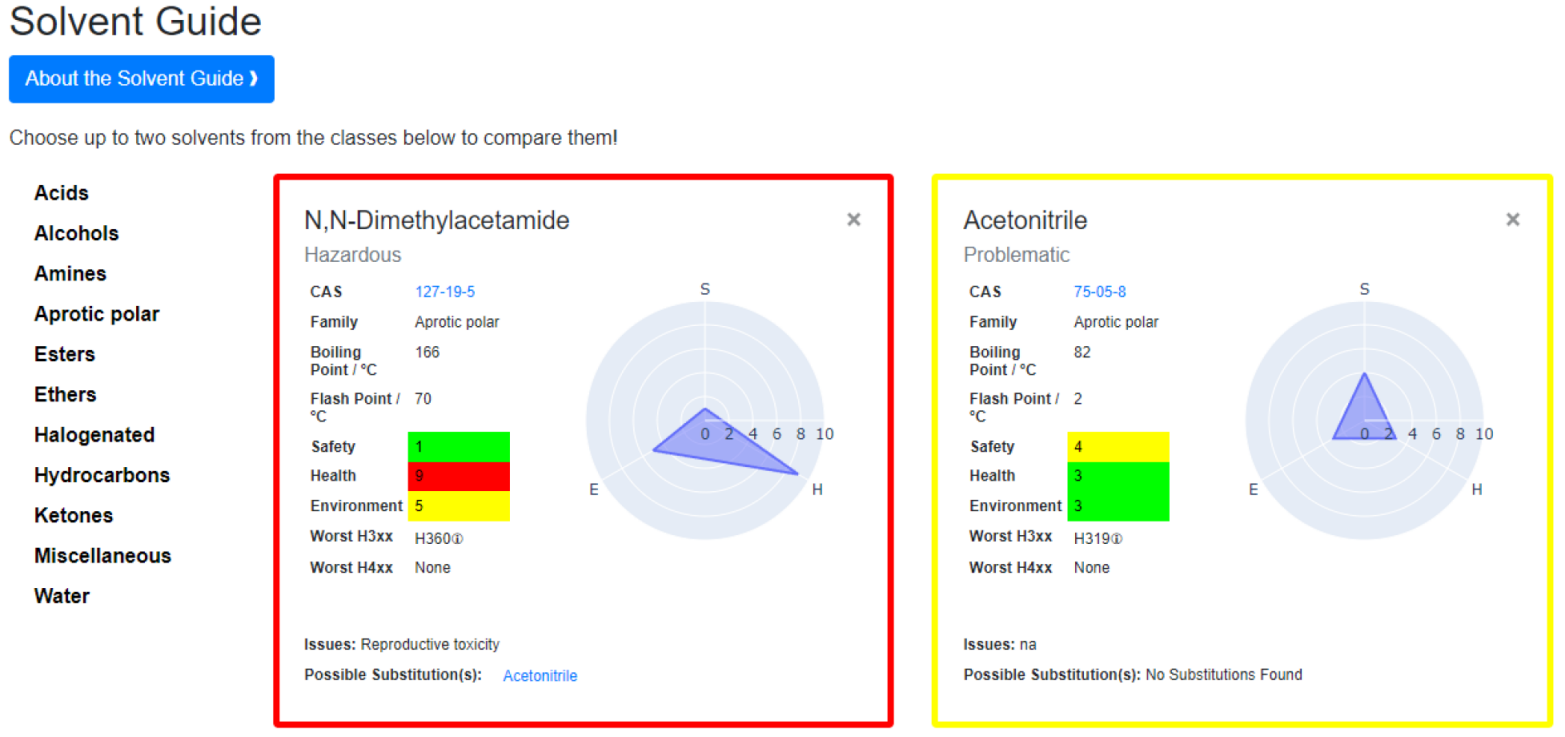
Solvent Guide where users can explore different solvent options for their reaction.

AI4Green is open-source, and adding new sustainable auxiliary applications is simple. The Python Flask backend built around blueprints gives new developers a low barrier. An example of an add-on is the Solvent Guide (Figure 6), which is a series of solvent flashcards. This can be accessed directly from the top navigation banner or as a user intervention while building a reaction. As previously discussed, the CHEM21 sustainability rating is displayed to the user when a solvent is selected. At this point, the user can open the solvent guide with this preloaded solvent flashcard. A second solvent can be selected for a side-by-side comparison. These solvent flashcards were created using data from the CHEM21 project, where the overall sustainability of the solvent is shown using the four-tier system. This is accompanied by a breakdown of the solvent’s health, safety, and environment scores, with each category given a score out of 10 and a corresponding hazard color. The full methodology for calculating these scores is described in the CHEM21 publication.^25^ The CAS number, linked to the PubChem entry for the solvent, is displayed to allow the user to access more information about the solvent. The family of the solvent, boiling point, flash point, and worst hazards are also displayed to guide the user further. For some solvents, a possible substitution is suggested based on an industrial solvent replacement guide and the reason for this substitution.^28^ The solvent guide aims to bring the most relevant information to the user on a single page, allowing the solvents to be easily compared side-by-side. This can empower the user to choose a more sustainable and suitable solvent for their reaction at the planning stage. Future add-on applications could include a solvent map for solvent substitution, solvent or reaction conditions prediction for a specific reaction, life cycle analysis of reactions, and retrosynthesis for a target product.

### User Feedback and Application Development

AI4Green has a growing userbase and is currently used by several academic groups, primarily at the University of Nottingham. As the application has evolved, users have had a direct input into new features either by contacting the team directly or by attending our regular user group meetings. Early feedback identified that the ELN must be easy to use, convenient for the laboratory chemist, and allow data to be easily shared throughout the group. Specific examples of features added because of user feedback include an automatic unique numbering system for reactions on their creation since users identified that, in a busy lab schedule, several reactions may be worked on concurrently; an autosave feature to ensure data were not accidentally lost if the browser tab is closed without saving; and adding reagents and solvents by CAS number, since this is a common identifier for laboratory chemists.

## Conclusion

In this work we present an open-source web application, AI4Green, which combines the practical benefits of an ELN alongside a framework for encouraging green and sustainable chemistry. Despite the manifold benefits, many academic groups are yet to adopt usage of an ELN. AI4Green provides an accessible platform for chemists to store and share their research and receive feedback on the sustainability of their reactions. Research teams can be easily organized into Workgroups and Workbooks, with different levels of permission for different user types. The Reaction Builder provides a semiautomated route to generating sustainability, hazard reports, and risk assessments. The Solvent Guide is an example of an add-on application to encourage sustainable solvent selection. However, there are still many features to implement in AI4Green. Data sharing between different Workgroups or to the public is not yet possible. We also aim to use inputted reaction data to make intelligent sustainability suggestions. This may be to suggest using a less hazardous solvent or reagent, predict milder suitable reaction conditions, or simulate Life Cycle Analysis for process scale-up. There will be challenges in preserving data privacy for users, who will be able to decide whether to share data fully, partially, or not at all. Additional features to be implemented include searching reactions by component name or a substructure, a mechanism for PIs to approve reactions, and a sustainability dashboard to allow groups to set sustainability goals and track their progress toward them. AI4Green provides an exciting initial framework to unite an ELN with sustainable chemistry. It has a growing user base and rapidly evolving functionality.

## Data Availability

AI4Green is open-source and released under the AGPL-3.0 license. Full source code, installation instructions and links to our video tutorials and user guides can be found at https://github.com/AI4Green/AI4Green.

## Supporting Information Available

Further detail on the database schema (PDF)

## ABBREVIATIONS

AJAX: Asynchronous JavaScript and XML
CAS: Chemical Abstracts Service
CSS: Cascaded Style Sheets
CSV: Comma-Separated Values
ECHA: European Chemicals Agency
ELN: Electronic Laboratory Notebook
GHS: Globally Harmonized System
HTML: HyperText Markup Language
JS: JavaScript
pdf: Laboratory Chemical Safety Sheets
LCSS: Portable Document Format
RXSMILES: Reaction SMILES
SMILES: Simplified Molecular-Input Line-Entry System
