# Supplementary material for: AI4Green: An Open-Source ELN for Green and Sustainable Chemistry
Source: J Chem Inf Model. 2023 May 8;63(10):2895–901. doi: 10.1021/acs.jcim.3c00306 (PMC10207257; doi:10.1021/acs.jcim.3c00306)
Supplement: Supplementary file 1 — ci3c00306_si_001.pdf [file ci3c00306_si_001.pdf]

Supporting Information

AI4Green: An Open-Source ELN for Green and Sustainable Chemistry

Samuel Boobier,[a] *Joseph C. Davies,*[a] *Ivan Derbenev,*[b] *Christopher M. Handley*[b] *and Jonathan D. Hirst*[a] *

[a]School of Chemistry, University of Nottingham, University Park, Nottingham, NG7 2RD, United Kingdom

[b]Digital Research Service, University of Nottingham, Jubilee Campus, Nottingham, NG8 1BB, United Kingdom

*Jonathan D. Hirst – School of Chemistry, University of Nottingham, University Park, Nottingham, NG7 2RD; Email: jonathan.hirst@nottingham.ac.uk

Database Schema

AI4Green is implemented with a single relational database using either SQLite (locally) or Postgres (Cloud hosted). This database consists of a series of linked tables as shown in **Figure S1**. Tables are either directly linked (one-to-one) or an additional table is made to accommodate many-to-one relationships (e.g., a Workgroup can have more than one Person). We make a distinction between a User and a Person. The User table contains personal information such as username, full name, and email, and is linked to the Role table (to specify whether the user has admin privileges). Each User is linked to a Person, which in turn is linked to their Workgroups (note there are three Person_Workgroup tables for each of Principal Investigator, Senior Researcher, and Standard Member), Workbooks, Requests, and Notifications. This allows a User to delete their profile and all their identifying information while retaining any reaction data for the rest of the Workgroup. Data from PubChem is

populated into the Compound table when the database is initially set up. New Compound data is also added periodically to add newly added PubChem data. The Compound table is linked CompoundErrorReport table to allow users to flag any information from PubChem which may be incorrect. There is also a NovelCompound table, which allows users to input their own compound data. This is linked to the Workgroup, ensuring only members of this Workgroup have access to this potentially private data. The Reaction table contains information about the reaction itself and is linked to a Person (creator) and a Workgroup, again maintaining the privacy of that data. The time of creation, time of update, and status (active or locked) give increased data integrity through timestamping. The Institution table (not fully implemented to date) could be used to separate Workgroups based on institution and tailor AI4Green for their specific needs. The Reaction_Reaction table is also yet to be implemented and will allow users to link a new reaction to previous reactions (for example, the same reaction run at different conditions). Finally, there are several referencing table providing details about hazard code sentences, solvents, news items (which are shown on the home page), and elements (used to calculate the sustainability of the chemical elements used in the reactions).

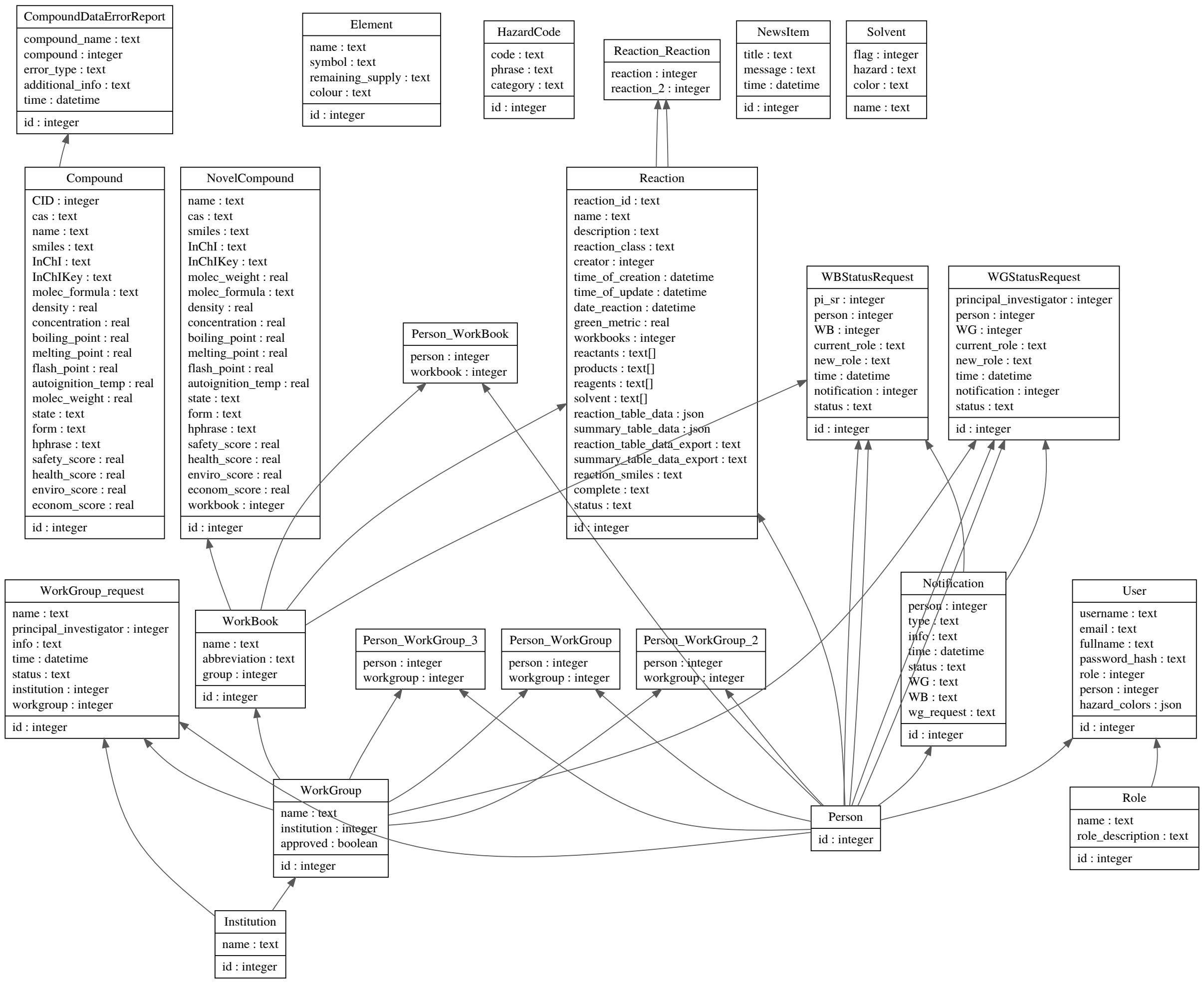

Figure S1. The different tables used in the relational database implemented in AI4Green.
